# Regulation of Biofilm Exopolysaccharide Production by Cyclic Di-Guanosine Monophosphate

**DOI:** 10.3389/fmicb.2021.730980

**Published:** 2021-09-10

**Authors:** Myles B. Poulin, Laura L. Kuperman

**Affiliations:** Department of Chemistry and Biochemistry, University of Maryland, College Park, College Park, MD, United States

**Keywords:** biofilm, exopolysaccharide, cyclic di-guanosine monophosphate, cellulose, alginate, Pel polysaccharide, PNAG

## Abstract

Many bacterial species in nature possess the ability to transition into a sessile lifestyle and aggregate into cohesive colonies, known as biofilms. Within a biofilm, bacterial cells are encapsulated within an extracellular polymeric substance (EPS) comprised of polysaccharides, proteins, nucleic acids, lipids, and other small molecules. The transition from planktonic growth to the biofilm lifecycle provides numerous benefits to bacteria, such as facilitating adherence to abiotic surfaces, evasion of a host immune system, and resistance to common antibiotics. As a result, biofilm-forming bacteria contribute to 65% of infections in humans, and substantially increase the energy and time required for treatment and recovery. Several biofilm specific exopolysaccharides, including cellulose, alginate, Pel polysaccharide, and poly-*N*-acetylglucosamine (PNAG), have been shown to play an important role in bacterial biofilm formation and their production is strongly correlated with pathogenicity and virulence. In many bacteria the biosynthetic machineries required for assembly of these exopolysaccharides are regulated by common signaling molecules, with the second messenger cyclic di-guanosine monophosphate (c*-*di-GMP) playing an especially important role in the post-translational activation of exopolysaccharide biosynthesis. Research on treatments of antibiotic-resistant and biofilm-forming bacteria through direct targeting of c-di-GMP signaling has shown promise, including peptide-based treatments that sequester intracellular c-di-GMP. In this review, we will examine the direct role c-di-GMP plays in the biosynthesis and export of biofilm exopolysaccharides with a focus on the mechanism of post-translational activation of these pathways, as well as describe novel approaches to inhibit biofilm formation through direct targeting of c-di-GMP.

## Introduction

Bacterial biofilm formation is a pervasive lifestyle adaptation that confers the organism with resistance to environmental stress, provides protection against antibiotics, and enables evasion of the host immune defenses (Costerton et al., 1995, 1999; Vuong et al., 2004b; Vu et al., 2009). As a result, bacterial biofilms are particularly pervasive in chronic and hospital acquired infections (Costerton et al., 1999). Bacterial biofilms consist of sessile bacterial communities embedded within a self-produced extracellular polymeric substance (EPS) composed of exported polysaccharides, proteins, nucleic acids, lipids, and small molecules (Flemming et al., 2007; Flemming and Wingender, 2010; Joo and Otto, 2012). The EPS serves to facilitate cell-cell adhesion, surface attachment, and acts as a physical barrier to protect the enclosed bacterial community from environmental stress. Treating infections resulting from bacterial biofilms remains a major challenge due to the altered metabolism of the bacteria, natural antibiotic resistance, and immune system evasion conferred by the protective biofilm EPS (Costerton et al., 1995, 1999; Vuong et al., 2004b; Singh et al., 2017; Yan and Bassler, 2019). Blocking EPS production or disrupting existing EPS components has been shown to prevent biofilm formation and increase susceptibility to antibiotic treatment (Gunn et al., 2016). Thus, a detailed understanding the molecular mechanisms of biofilm EPS production is essential for the development of new anti-biofilm therapeutics to specifically target these pathways.

**FIGURE 1:**
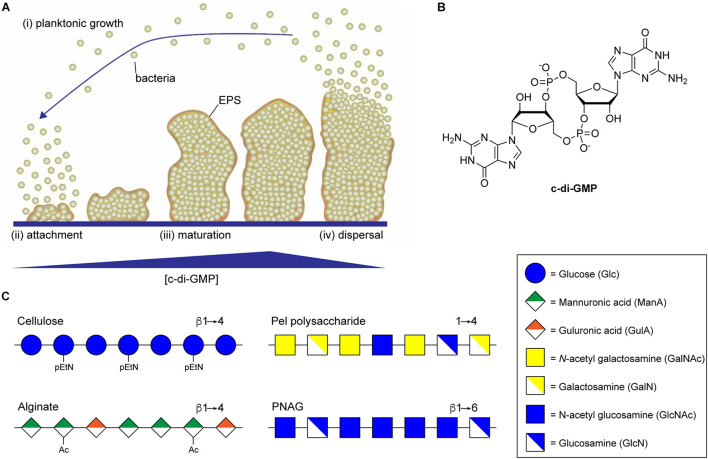
Biofilm formation is regulated by c-di-GMP. **(A)** Biofilm development begins when planktonic bacteria (*i*) attach to a surface through the action of specific adhesins or non-specific surface hydrophobicity (*ii*). Increasing intracellular concentrations of c-di-GMP then result in secretion of biofilm EPS components, including exopolysaccharide, leading to the maturation of complex three-dimensional biofilm microstructures (*iii*). Eventually, decreasing concentrations of c-di-GMP lead to the production of dispersal agents, surfactants and cell motility factors resulting in dispersal of planktonic bacterial from the biofilm (*iv*). **(B)** Structure of the di-nucleotide second messenger, c-di-GMP. **(C)** Representative structures of the biofilm exopolysaccharides cellulose, alginate, Pel, and PNAG.

In most bacteria, the switch from planktonic growth to the sessile biofilm lifestyle is induced in response to a common second messenger cyclic di-guanosine monophosphate (c-di-GMP) (Simm et al., 2004; Hengge et al., 2016; Yoon and Waters, 2021). Responses to the intracellular concentration of c-di-GMP have been implicated in all phases of biofilm formation from initial attachment, through proliferation, and dispersal (Figure 1A; Davey and O’Toole, 2000; Joo and Otto, 2012), and promote the production of numerous EPS components involved in biofilm assembly, including extracellular DNA, protein adhesin, and exopolysaccharide (Römling, 2012). Intracellular levels of c-di-GMP, whose structure is shown in Figure 1B, are regulated by diguanylate cyclase (DGC) and phosphodiesterase (PDE) enzymes that catalyze the synthesis and breakdown of the second messenger, respectively (Tal et al., 1998). DGC enzymes typically contain a GGDEF domain responsible for c-di-GMP synthesis through the cyclization of two equivalents of guanosine triphosphate (GTP) (Dahlstrom and O’Toole, 2016), and a sensory domain that activates the DGC activity in response to external stimuli, such as nutrient concentrations (Zähringer et al., 2013), temperature (Almblad et al., 2021), or phosphorylation (Teixeira et al., 2021). PDEs, on the other hand, typically contain EAL or HD-GYP domains that catalyze the hydrolysis of c-di-GMP to produce a linear 5′-phosphoguanylyl-(3′-5′)-guanosine (pGpG) dinucleotide product (Ryjenkov et al., 2005; Schmidt et al., 2005). Many bacteria contain multiple DGCs and PDEs that contribute to the regulation of intracellular c-di-GMP, making it challenging to study the impact of individual DGCs and PDEs on c-di-GMP levels and on the many pathways c-di-GMP regulates (Ren et al., 2016).

Since the discovery of c-di-GMP, additional cyclic-di-nucleotides, including cyclic-di-adenosine monophosphate (c-di-AMP) and the mixed nucleotide second messenger cyclic-adenosine monophosphate-guanosine monophosphate (c-AMP-GMP), have been identified (Witte et al., 2008; Corrigan et al., 2011; Davies et al., 2012; Dias da et al., 2020; Xiong et al., 2020; Yoon and Waters, 2021). C-di-AMP was originally identified in *Bacillus subtilis* where it was shown to play a role in sporulation (Witte et al., 2008), and has since been identified in other Gram-positive bacteria including *Staphylococcus aureus*, where it is implicated in controlling cell size (Corrigan et al., 2011), and *Streptococcus mutans*, where it is implicated in the regulation of glucan biosynthesis (Cheng et al., 2015; Peng et al., 2016). C-AMP-GMP, on the other hand, was first identified in *Vibrio cholera* where it appears to regulate chemotaxis and promotes intestinal colonization (Davies et al., 2012). Like c-di-GMP, both c-di-AMP, and c-AMP-GMP have been suggested to function in regulating the transition from planktonic growth to sessile biofilm lifestyle (Hengge et al., 2016; Xiong et al., 2020), though the specific mechanisms by which c-di-AMP and c-AMP-GMP function are less well understood. Thus, for this review we will specifically focus on mechanisms by which c-di-GMP regulates exopolysaccharide biosynthesis during biofilm formation.

The primary mechanism by which c-di-GMP regulates cell cycle progression, bacterial motility, and biofilm formation involves the interaction of c-di-GMP with a wide assortment of effectors, including riboswitches, DNA-binding proteins, protein complexes, and enzymes whose activities are allosterically regulated through binding of c-di-GMP (Valentini and Filloux, 2019). Several recent reviews thoroughly describe how c-di-GMP binding to these effectors influences transcriptional regulation, signaling, and bacterial virulence (Valentini and Filloux, 2019; Yi et al., 2019; Kunz and Graumann, 2020; Ma et al., 2020; Yoon and Waters, 2021). This review will specifically focus on how c-di-GMP post-translationally regulates biofilm formation through allosteric activation of enzyme complexes involved in the biosynthesis and export of biofilm exopolysaccharides that serve as key structural components of the biofilm EPS.

## Structure and Function of Common Biofilm Exopolysaccharides

Large, secreted exopolysaccharides are among the most abundant components of bacterial biofilm EPS (Ghafoor et al., 2011; Maunders and Welch, 2017; Cugini et al., 2019). Exopolysaccharide production is also highly correlated with biofilm formation, and disruption of genes involved in exopolysaccharide production results in the inability of bacteria to form a mature biofilm (Watnick and Kolter, 1999; Danese et al., 2000; Friedman and Kolter, 2003, 2004; Agladze et al., 2005). Polysaccharides are key components of biofilm EPS and their composition can vary depending on bacterial species, environmental factors, and the stage of the biofilm lifecycle (Wozniak et al., 2015). The specific biofilm polysaccharides can also vary between strains of the same bacteria, for example biofilm formation in *Pseudomonas aeruginosa* PAO1 depends primarily on Psl polysaccharide while biofilms of the PA14 strain are Pel polysaccharide dependent (Friedman and Kolter, 2004; Colvin et al., 2011; Ghafoor et al., 2011). A number of different types of structural polysaccharides can contribute to biofilm formation, including lipopolysaccharides (Chatterjee and Chaudhuri, 2006), capsular polysaccharides (Fabretti and Huebner, 2005), and wall teichoic acids (Rajagopal and Walker, 2016), while others may actually inhibit biofilm formation (Nakao et al., 2012; Ruhal et al., 2015). In addition to these polysaccharides, a small number of secreted exopolysaccharide biosynthetic loci are highly correlated with biofilm formation and are conserved amongst diverse bacterial species (Bundalovic-Torma et al., 2020). These include pathways encoding for biosynthesis of bacterial cellulose (Ross et al., 1991), alginate (Ryder et al., 2007), Pel polysaccharide (Friedman and Kolter, 2004), and poly-*N*-acetylglucosamine (PNAG) (Heilmann et al., 1996; Arciola et al., 2015; Figure 1C). Each of these exopolysaccharides directly contributes to biofilm formation and function as major virulence factors for their respective organisms (Vuong et al., 2004a, b; Ghafoor et al., 2011; Koo et al., 2013; Tian et al., 2013). Multiple mechanisms regulate exopolysaccharide biosynthesis, including transcriptional regulation and quorum sensing pathways (O’Toole and Kolter, 1998; Jackson et al., 2002; Wang et al., 2005; Jonas et al., 2008; Laverty et al., 2013, 2014), and these can differ substantially by organism. One thing all of these exopolysaccharides have in common is that their synthesis is catalyzed through the action of processive glycosylsynthase enzyme complexes whose activity is directly regulated through binding to c-di-GMP (Mack et al., 1996; Friedman and Kolter, 2004; Low and Howell, 2018).

### Cellulose Biosynthesis

Cellulose, a polymer of glucose (Glc) units linked through β-(1→4) glycosidic bonds (O’Sullivan, 1997), is the most abundant polymer on Earth and is produced by an abundance of plants, algae, and prokaryotes (Ross et al., 1987). Within prokaryotes, cellulose production has been observed in both Gram-positive and Gram-negative bacteria and is present in the secreted EPS matrix of biofilm-producing bacteria (Zogaj et al., 2001; Serra et al., 2013; Römling and Galperin, 2015). The cellulose present in bacterial biofilms is also frequently decorated with additional chemical modifications, such as *O*-acetates (Römling and Galperin, 2015) and *O*-phosphatidylethanolamine (pEtN) groups (Thongsomboon et al., 2018), that are introduced onto the growing polysaccharide during its biosynthesis and secretion (Sun et al., 2018; Anderson et al., 2020). Bacterial cellulose biosynthesis and secretion is highly dependent on cellular c-di-GMP concentration (Ross et al., 1990). In fact, studies of cellulose biogenesis in the Gram-negative soil bacterium *Komagataeibacter xylinus* (formerly *Acetobacter xylinum*) resulted in the identification of c-di-GMP as a bacterial second messenger by Moshe Benziman’s laboratory in 1987 (Ross et al., 1987). *In vitro*, the presence of c-di-GMP can increase cellulose biosynthesis by membrane preparations of *K. xylinus* by between 50 and 200-fold (Ross et al., 1990). Although there is some evidence that c-di-GMP may impact cellulose biogenesis at the transcriptional level through binding and activation of the cellulose synthase promoter (Fazli et al., 2011), the predominant mechanism of activation in *K. xylinus* appears to be through direct allosteric activation of the cellulose synthase complex itself.

**FIGURE 2:**
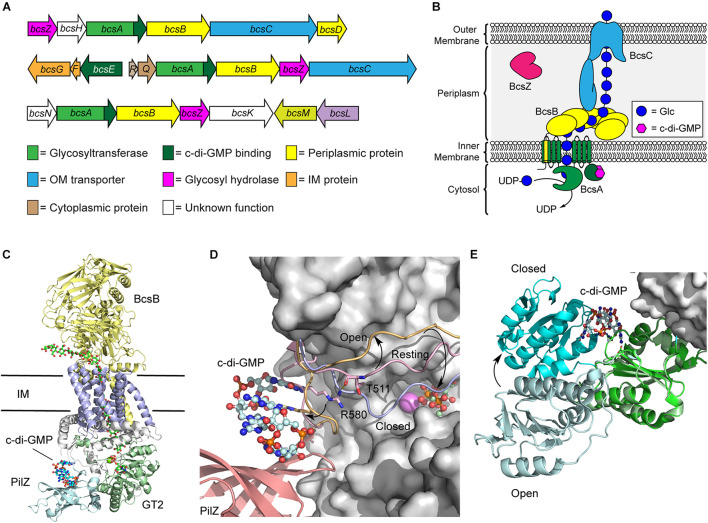
Regulation of the biosynthetic machinery for cellulose biogenesis by c-di-GMP. **(A)** Representative *Bcs* operons from *K. xylinus* (top), *E. coli* (middle), and *Agrobacterium fabrum* (bottom) encoding the enzymes required for cellulose biogenesis. **(B)** Organization of the biosynthetic machinery of the cellulose synthase of *K. xylinus*. **(C)** Crystal structure of the BscAB complex of *Rhodobacter spharoides* bound to c-di-GMP and UDP (PDB id 4P00). BcaB is shown in yellow, the BcsA transmembrane domains, GT2 domain, and PilZ domain are shown in blue, green and cyan, respectively. **(D)** C-di-GMP binding to the PilZ domain of BcsA results in a conformational change of the “gating loop” allowing access for UDP-Glc to bind to the active site. The conformation of the “gating loop” in the resting (pink, PDB id 4HG6), open c-di-GMP bound (orange, PDB id 4P02) and closed UDP bound (blue, PDB id 4P00) conformations. **(E)** Structure of BcsE bound to dimeric c-di-GMP and the BcsQR complex (PDB id 6YBB) compared to the structure of BcsE with monomeric c-di-GMP (PDB id 6TJ0). The BcsQR structure is shown as a white surface, the *N*-proximal domain (cyan) and *C*-proximal domain (green) of BcsE bound to dimeric c-di-GMP.

Soon after the discovery that cellulose biogenesis in bacteria is dependent on c-di-GMP, the first bacterial cellulose synthase operon from *K. xylinus* was identified in 1990 (Wong et al., 1990) and found to encode four core proteins (BcsA, BcsB, BcsC, and BcsD) required for cellulose biosynthesis in Gram-negative bacteria. Variants of this operon have since been identified in other Gram-negative and Gram-positive bacteria (Figure 2A), including domesticated *Escherichia coli* K12 strains, where the operon is inactive as the result of a premature stop codon in the *bcsQ* gene (Römling and Galperin, 2015). Bacterial cellulose synthesis operons can be divided into three major classifications and divided into subtypes depending on the order and presence of genes in these operons (Römling and Galperin, 2015; Figure 2A). Two proteins encoded by this operon, BcsA and BcsB, interact together to form the bacterial cellulose synthase (Bcs) enzyme (Ross et al., 1991), and is the minimum structure required for cellulose synthesis *in vitro* (Omadjela et al., 2013). The remaining proteins, BcsC and BcsD, are required for cellulose polymerization and secretion *in vivo* (Saxena et al., 1994).

BcsA and BcsB typically exist as separate polypeptide chains, though a single polypeptide fusion has been observed in some bacterial species with no impact on cellulose production (Saxena et al., 1994; Umeda et al., 1999). The BcsA protein contains the primary catalytic domains for both polymerization of cellulose using UDP-Glc as a precursor and membrane transport (Figure 2B; Morgan et al., 2013; Omadjela et al., 2013). It is composed of two transmembrane domains, that localize to the bacterial inner membrane, flanking a cytosolic glycosyltransferase family 2 (GT2) domain, responsible for cellulose polymerization, and followed by a *C*-terminal PilZ domain that interacts directly with c-di-GMP (Morgan et al., 2014). PilZ domains were the first protein structural motifs identified that contain c-di-GMP binding motifs (Amikam and Galperin, 2006; Ryjenkov et al., 2006). They adopt a six stranded β-barrel topology and interact with intercalated dimeric c-di-GMP through conserved RXXXR and DXSXXG motifs (Amikam and Galperin, 2006; Benach et al., 2007). BcsB, conversely, is predominantly localized to the periplasm and anchored into the inner membrane via a *C*-terminal transmembrane domain that interacts with and stabilizes BcsA (Figure 2C; Morgan et al., 2013; Omadjela et al., 2013). Recent cryo-electron microscopy (cryo-EM) structures of the *E. coli* cellulose synthase complex show that BcsB assembles into a hexamer in the inner membrane that may serve to guide the growing cellulose polysaccharide through the periplasm and serve as a scaffold for binding of additional biosynthetic enzymes like BcsG, responsible for introducing pEtN modifications onto the growing polysaccharide (Krasteva et al., 2017; Abidi et al., 2021; Acheson et al., 2021).

The predominant mechanism by which c-di-GMP regulates cellulose biosynthesis is through the allosteric activation of the BcsA protein. The *C*-terminal PilZ domain of BcsA binds directly to dimeric c-di-GMP through its conserved RXXXR Motif (Morgan et al., 2014, 2016). In the absence of c-di-GMP binding, BscA adopts an auto-inhibited conformation in which a gating loop (Figure 2D) sits over the BcsA active site, preventing access to the substrate, UDP-Glc (Morgan et al., 2013). Binding of the second messenger to the Pilz domain of BcsA induces a conformational change in the gating loop, granting UDP-Glc access to the active site (Morgan et al., 2014, 2016). Correspondingly, higher intracellular c-di-GMP levels correlate with increased BcsA activity, and thus encourage more cellulose production, as well as production of longer cellulose polymers (Omadjela et al., 2013; Richter et al., 2020).

More recently, it was discovered that c-di-GMP also binds with another Bcs protein, BcsE (Fang et al., 2014), which is present in operons encoding for pEtN cellulose production (Thongsomboon et al., 2018). The presence of BcsE significantly increases cellulose secretion *in vivo* and is thought to aid in formation and stabilization of the cellulose synthase inner membrane complex (Krasteva et al., 2017). The BcsE protein of *E. coli* is a 59 kDa soluble protein found in the cytoplasm that consists largely of a 313-aa domain of unknown function (DUF2819) preceded by a 161-aa long *N*-terminal domain (Fang et al., 2014). The structure of BcsE, determined by Petrya Krasteva’s laboratory in 2020 (Zouhir et al., 2020), revealed that the *C*-proximal DUF2819 domain closely resembles a degenerate GGDEF domain. GGDEF domains are commonly found in DGC and PDE enzymes that catalyze the biosynthesis and breakdown of c-di-GMP (Ryjenkov et al., 2005; Schmidt et al., 2005). The GGDEF domain of BcsE is missing key catalytic residues for DGC activity but maintains c-di-GMP binding through a conserved I-site motif (Zouhir et al., 2020; Abidi et al., 2021). C-di-GMP binding to BcsE also induces a conformational change that places the *C*-proximal domain in closer proximity to the *N*-proximal domain (Figure 2E).

The BcsE protein forms a 2:2:2 complex with BcsQ and BcsR that is in turn recruited to the cellulose synthase complex through interactions with the PilZ domain of BcsA (Zouhir et al., 2020; Abidi et al., 2021; Acheson et al., 2021). The close binding of BcsE with the PilZ domain of BcsA leads to the hypothesis that the soluble BcsE protein serves to sequester cytosolic c-di-GMP and deliver it to the PilZ domain of BcsA to facilitate the processive polymerization of cellulose (Zouhir et al., 2020), though detailed experimental evidence to support this hypothesis is lacking. Interestingly, BcsE is encoded within a small three gene *bcsEFG* operon in *E. coli* and located on the opposite strand of the larger *bcsRQABZC* operon that encodes the larger cellulose synthase complex (Römling and Galperin, 2015). The BcsG protein functions as a phosphoethanolamine transferase introducing the pEtN modifications present on ∼50% of the Glc units of *E. coli* cellulose (Sun et al., 2018; Thongsomboon et al., 2018; Anderson et al., 2020). The proximity of the genes encoding for BcsE, BcsF, and BcsG raise the intriguing possibility that c-di-GMP binding to BcsE may regulate the pEtN transferase activity of BcsG through the formation of a BcsEFG complex (Thongsomboon et al., 2018). This would signify that c-di-GMP plays a dual role in activating cellulose synthesis and regulating its pEtN modification, although additional experimental support for this model is still needed.

Significant strides have been made to understand the mechanism of cellulose synthase activation by c-di-GMP in the three decades since the discovery of this important bacterial second messenger but given the complexity of the cellulose biosynthetic machinery there is still the need for more details on how the biogenesis of this important polysaccharide is regulated. Specifically, does c-di-GMP regulate the modification of cellulose with pEtN in *E. coli* and other Gram-negative bacteria, and if so, what are the molecular details underlying this mechanism of this regulation?

### Alginate Biosynthesis

**FIGURE 3:**
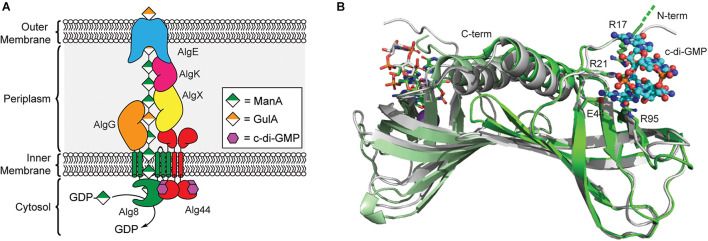
Regulation of alginate biosynthesis through the binding of c-di-GMP to Alg44. **(A)** Organization of the biosynthetic machinery of alginate synthase in *P. aeruginosa*. **(B)** Binding of c-di-GMP dimer results in a small conformational change in the PilZ domain of Alg44. Structures of dimeric Alg44 PilZ domain bound to c-di-GMP dimer (green, PDB id 4RT0) or a Alg44 R95A mutant bound to c-di-GMP monomer (white, PDB id 4RT1).

The first biofilm exopolysaccharide of *P. aeruginosa* discovered was alginate, and it remains the best-studied of the three primary *P. aeruginosa* biofilm polysaccharides due to its prominent role in cystic fibrosis (Evans and Linker, 1973; Pedersen et al., 1990; Remminghorst and Rehm, 2006b). This linear exopolysaccharide consists of non-repeating *O*-acetylated (1→4)-linked residues of β-d-mannuronic acid (ManA) and α-l-guluronic acid (GulA) (Remminghorst and Rehm, 2006b). The biosynthetic machinery for alginate secretion is comprised of a multiprotein complex spanning the bacterial cell envelope that requires the cooperation of thirteen proteins (Figure 3A; Rehman et al., 2013). Twelve of the proteins are encoded by the *alg* operon (Chitnis and Ohman, 1993), while *algC*, which encodes a phosphomannomutase required for biosynthesis of the GDP-ManA sugar nucleotide building block for alginate production, is located elsewhere in the genome (Zielinski et al., 1991).

In 2007, Stephen Lory’s laboratory was the first to report that alginate biogenesis in *P. aeruginosa* was dependent on c-di-GMP (Merighi et al., 2007) after it was shown that the *alg44* gene encodes for a PilZ domain containing protein (Amikam and Galperin, 2006; Remminghorst and Rehm, 2006a). The *N*-terminal PilZ domain of Alg44 is connected to a periplasmic *C*-terminal membrane fusion domain (Dinh et al., 1994) via a single pass transmembrane helix (Merighi et al., 2007; Whitney et al., 2015). Results from bacterial two-hybrid assays and chemical crosslinking show that Alg44 directly interacts with Alg8 in the inner membrane to form the alginate polymerase complex (Moradali et al., 2015) and to a periplasmic scaffold made up of AlgK, AlgE, AlgG, and AlgX (Rehman et al., 2013), which assist in the translocation of the poly-ManA chain across the periplasm (Franklin et al., 2011). Alg8 functions as the polymerase for alginate biogenesis and is an inner membrane protein containing five transmembrane helices and a cytoplasmic GT2 domain similar to that of cellulose synthase enzyme BcsA (Remminghorst and Rehm, 2006c; Oglesby et al., 2008; Remminghorst et al., 2009). It is through the interaction of Alg44 with Alg8 that c-di-GMP is presumably able to post-translationally regulate production of alginate in *P. aeruginosa*, though exact details on the mechanism of this activation will require structure determination of the Alg44/Alg8 complex.

A crystal structure of the Alg44 PilZ domain bound to c-di-GMP was determined in 2015 by P. Lynne Howell’s laboratory (Whitney et al., 2015). This model reveals that the Alg44 PilZ domain exists as a homodimer (Figure 3B) with each PilZ domain bound to dimeric c-di-GMP through the canonical RXXXR motif. *In vitro* data indicates that dimerization of Alg44 (Whitney et al., 2015) and its interactions with Alg8 (Moradali et al., 2015) both occurs even in the absence of c-di-GMP. Dimerization of Alg44 has also been observed for the full-length protein (Moradali et al., 2017), although it is not clear how dimerization might impact its interactions with Alg8. Both R21 and E44 of Alg44 are essential for c-di-GMP binding, while R17 and R95 are required for binding to the dimeric second messenger (Whitney et al., 2015). There is also evidence that residues in the *C*-terminal domain of Alg8, specifically H323, T457, and E460, contribute to the c-di-GMP-dependent activation of the Alg44/Alg8 complex (Moradali et al., 2015, 2017). There is some evidence that binding of c-di-GMP to Alg44 induces a conformational change in the PilZ dimer (Whitney et al., 2015) that may enable the activation of Alg8 in a mechanism similar to cellulose synthase, although further studies are required to test this hypothesis. Mutations of Alg44 that block c-di-GMP binding also prevent the production of alginate in *P. aeruginosa* (Merighi et al., 2007), thus, inhibiting the binding of c-di-GMP to Alg44 may be an effective strategy to treat cystic fibrosis patients suffering from persistent *P. aeruginosa* infections.

### Pel Polysaccharide Biosynthesis

The Pel polysaccharide is the second of three primary biofilm exopolysaccharides that contribute to biofilm formation in *P. aeruginosa* (Ryder et al., 2007; Ghafoor et al., 2011). While alginate primarily serves a protective role in mature biofilms of mucoid *P. aeruginosa*, the Pel polysaccharide was first identified for its role in the formation of pellicle biofilms at the air-liquid interface in non-mucoid strains of *P. aeruginosa* (Friedman and Kolter, 2003). Originally thought to be a glucose-rich polysaccharide with a composition similar to cellulose (Friedman and Kolter, 2003, 2004), more recent analysis of Pel found that it consists primarily of *N*-acetylgalactosamine (GalNAc) and *N*-acetylglucosamine (GlcNAc) residues connected through (1→4)-glycosidic linkages. Pel is also partially de-*N*-acetylated through the action of the carbohydrate esterase PelA during Pel biogenesis and secretion (Colvin et al., 2013; Jennings et al., 2015). This cationic Pel polysaccharide plays a role in cell adhesion and cross-links extracellular DNA in the periphery and stalk region of mature *P. aeruginosa* biofilms (Jennings et al., 2015). Although the *pel* operon was initially identified in *P. aeruginosa*, homologous loci have since been identified in diverse Gram-negative and Gram-positive bacterial species (Bundalovic-Torma et al., 2020; Whitfield et al., 2020a).

Like alginate and cellulose, Pel biogenesis is post-translationally regulated by c-di-GMP (Lee et al., 2007); however, unlike the previous exopolysaccharides, none of the proteins involved in Pel production contain a PilZ domain. By screening the proteins encoded in the *pel* operon for c-di-GMP binding, Stephen Lory’s lab identified that the cytoplasmic domain of PelD functions as a c-di-GMP binding proteins (Lee et al., 2007). PelD contains four predicted *N*-terminal transmembrane helices, and *C*-terminal GAF and a degenerate GGDEF domains. The structure of PelD’s *C*-terminal domains was reported by P. Lynne Howell’s laboratory in 2012 and revealed that dimeric c-di-GMP binds to a conserved RXXD motif present in the GGDEF domain of PelD (Whitney et al., 2012). In addition to R367 and D370 of the RXXD motif, R402 from the GGDEF domain is absolutely required for c-di-GMP binding, while R161 of the GAF domain interacts with bound c-di-GMP but is not essential for binding (Whitney et al., 2012). Moreover, the region linking the *N*-terminal transmembrane domain and *C*-terminal GAF/GGDEF domains of PelD is predicted to form a coiled-coil dimerization domain (Whitney et al., 2012). Experimental evidence for this dimerization and its importance in Pel production is still required.

**FIGURE 4:**
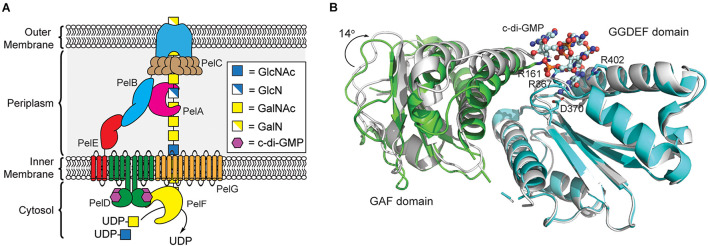
Pel polysaccharide biosynthesis is activated by c-di-GMP binding to the GGDEF domain of PelD. **(A)** Organization of the machinery for Pel polysaccharide biosynthesis in *P. aeruginosa*. **(B)** Binding of c-di-GMP dimer to the GGDEF domain (cyan) of PelD results in a 14° rotation of the GAF domain (green, PDB id 4DN0) relative to the structure of PelD in the absence of c-di-GMP (white, PDB id 4DMZ).

Currently, it is not clear exactly how c-di-GMP binding to PelD functions to post-translationally regulate Pel production. Recent results from bacterial two-hybrid assays and co-immunoprecipitation studies reported by P. Lynne Howell’s laboratory indicate that PelD forms an inner membrane complex with PelE and PelG that together recruit the glycosyltransferase PelF to the cell membrane (Figure 4A; Whitfield et al., 2020b). PelF is a soluble GT family 4 enzyme that is responsible for catalyzing the glycosyl transfer reaction required for Pel biosynthesis (Ghafoor et al., 2013). Interestingly, pure soluble PelF protein showed no catalytic activity *in vitro* but retained the ability to bind UDP, the presumed product of the glycosyl transfer reaction, with micromolar affinity (Jennings et al., 2015). This suggests that PelF recruitment to the inner membrane by the PelDEG complex may be required for PelF activity, although binding of c-di-GMP to PelD does not affect assembly of this complex (Whitfield et al., 2020b). A current hypothesis is that c-di-GMP binding to PelD induces a structural change in the PelDEFG quaternary structure leading to activation of Pel biogenesis. Structural studies of PelD have shown that a ∼14° shift in the position of the GAF domain relative to the GGDEF domain of PelD accompanies the binding of dimeric c-di-GMP (Figure 4B; Whitney et al., 2012) providing some evidence to support the conformational change model of Pel biosynthesis activation, although further structural analysis of the PelDEFG complex will be required to evaluate this hypothesis in detail.

### PNAG Biosynthesis

PNAG, also known as polysaccharide intercellular adhesin (PIA) in Gram-positive *Staphylococci* (Heilmann et al., 1996; Cramton et al., 1999), is one of the most common biofilm associated exopolysaccharides reported to date and produced by both Gram-positive and Gram-negative bacteria (Mack et al., 1994; Heilmann et al., 1996; Cramton et al., 1999; Wang et al., 2004; Izano et al., 2007; Hinnebusch and Erickson, 2008; Choi et al., 2009; Chen et al., 2014; Roux et al., 2015; Bundalovic-Torma et al., 2020). PNAG is a linear polysaccharide consisting of GlcNAc residues connected through β-(1→6) glycosidic linkages in which 15–20% of the GlcNAc residues are de-*N*-acetylated to glucosamine (GlcN), giving a cationic charge to the polysaccharide (Mack et al., 1996; Joyce et al., 2003; Sadovskaya et al., 2005). In addition, PNAG in *Staphylococcus epidermidis* and *Staphylococcus aureus* are decorated with *O*-succinate modifications on the 3-hydroxyl group of GlcNAc residues giving a zwitterionic, or sometimes even anionic, charge to the polysaccharide. Production of PNAG by these organisms is strongly associated with biofilm formation and virulence in animal disease models (Vuong et al., 2004a; Izano et al., 2007; Sloan et al., 2007; Nguyen et al., 2020).

Both biofilm formation and the production of PNAG in *E. coli* are dependent on c-di-GMP production (Boehm et al., 2009; Tagliabue et al., 2010), and this is believed to be the case in other Gram-negative bacteria as well. Interestingly, de-*N*-acetylated PNAG production in *S. epidermidis* is highly dependent on the GGDEF domain-containing protein, GdpS, but the mechanism by which GdpS regulates PNAG production is not dependent on c-di-GMP (Holland et al., 2008). In fact, GdpS is inactive as a diguanylate cyclase (Holland et al., 2008). The *gdpS* gene of *S. epidermidis* appears to regulate transcription of the *icaABCD* operon and biofilm formation through a mechanism that is independent of its protein coding function (Zhu et al., 2017). As it appears that PNAG biogenesis in Gram-positive *Staphylococcus* may occur independently of c-di-GMP regulation, here, we will focus on the role of c-di-GMP in post-translational regulation of PNAG production in Gram-negative bacteria.

**FIGURE 5:**
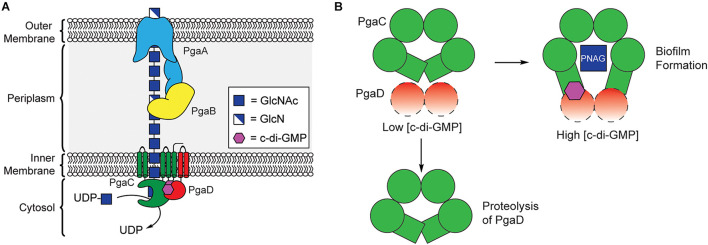
C-di-GMP regulates PNAG biosynthesis through stabilization of the PgaCD complex. **(A)** Organization of the biosynthetic machinery for PNAG biogenesis in *E. coli*. **(B)** A proposed model for allosteric activation of the PgaCD complex by c-di-GMP. The binding of c-di-GMP (purple) to the membrane proximal region of PgaC (green) and PgaD (red) results in stabilization of an active complex. Under low c-di-GMP conditions PgaD loosely associates with PgaC and is susceptible to protease degradation resulting in the inactivation of PgaC.

PNAG synthesis involves the cooperation of four proteins encoded by the *pgaAB*C*D* operon, collectively referred to as PNAG synthase (Figure 5A; Wang et al., 2004; Itoh et al., 2005). PgaC contains a GT2 domain, and acts as the primary biosynthetic enzyme in the production of PNAG, catalyzing the polymerization of GlcNAc from the precursor UDP-GlcNAc (Wang et al., 2004; Itoh et al., 2008). PgaC activity is highly dependent on the presence of PgaD, a small integral membrane protein that does not share any sequence homology to known protein folds, but is known to colocalize with PgaC in the bacterial inner membrane (Daley et al., 2005; Itoh et al., 2008). Despite evidence that PNAG production is dependent on c-di-GMP, none of the proteins encoded within the *pgaABCD* operon contain known c-di-GMP binding protein motifs.

In 2009, Urs Jenal’s laboratory showed that the concentration of PgaD protein in *E. coli* was dependent on the activity of the DGC enzyme YdeH, and that this regulation occurs post-translationally (Boehm et al., 2009). They later showed that PgaD is intrinsically unstable and rapidly degraded in the absence of c-di-GMP or PgaC (Figure 5B). They showed that c-di-GMP binds directly to both PgaC and PgaD to form a stable complex in the inner membrane (Steiner et al., 2013). Moreover, the binding of c-di-GMP to the PgaCD complex was shown to allosterically activate the glycosyltransferase activity of PgaC (Steiner et al., 2013). The nature of the complex between PgaCD and c-di-GMP and the exact mechanism by which it allosterically activates PgaC activity have yet to be determined and will likely require structure determination of this complex, but it is hypothesized that c-di-GMP binds in a membrane proximal pocket at the interface of the complex formed between PgaC and PgaD. The work of Urs Jenal is the first report of a c-di-GMP receptor relying on protein-protein interactions (Steiner et al., 2013) and may represent a new mechanism for c-di-GMP-dependent protein activation, although additional work is required to work out the mechanistic details of this activation mechanism.

## Targeted Biofilm Disruption by Interception of c-di-GMP

Infections by biofilm forming bacteria are common in chronic and hospital acquired infections. Infections by mucoid biofilm forming *P. aeruginosa*, for example, is commonly found in the lungs of cystic fibrosis patients (Pedersen et al., 1990; Koch and Høiby, 1993; Lund-Palau et al., 2016). Mucoid *P. aeruginosa* strains secrete large quantities of alginate, which exacerbates the symptoms of cystic fibrosis and makes the bacteria resistant to common antibiotic treatments. Given the central role of c-di-GMP signaling in biofilm formation, targeting it has emerged as an attractive approach for treating bacterial biofilm infections that are resistant to common antibiotic therapies (Römling et al., 2005). Much of the efforts to block c-di-GMP signaling have focused on the inhibition of DGC enzymes (Qvortrup et al., 2019; Cho et al., 2020). However, bacteria typically encode multiple DGCs that all contribute to c-di-GMP signaling and biofilm exopolysaccharide biogenesis, meaning that inhibition of individual DGC enzymes will likely be insufficient as an anti-biofilm strategy. More recent efforts to inhibit biofilm exopolysaccharide biosynthesis focus on inhibiting the binding of c-di-GMP to receptors, or by physically sequestering c-di-GMP inside the cell.

### Inhibition of c-di-GMP Binding to Alg44

In 2017, Vincent Lee’s lab used a differential radial capillary action of ligand assay (Roelofs et al., 2011) to screen for a chemically diverse library of compounds that inhibit the binding of the PilZ domain of Alg44 to ^32^P-c-di-GMP (Zhou et al., 2017). From this screening they identified thiol-benzo-triazolo-quinazolinone as a micromolar inhibitor of Alg44. This compound inhibits c-di-GMP binding through the covalent modification of C98 of Alg44 (Zhou et al., 2017), which results in a small but significant decrease in alginate production. After the identification of this thiol-benzo-triazolo-quinazolinone inhibitor of Alg44, Ebselen oxide and its analogs have also been shown to inhibit Alg44 binding to c-di-GMP via the covalent modification of the same C98 residue (Kim et al., 2021) to block alginate production without significantly inhibiting the growth of *P. aeruginosa*. Interestingly, the ability of Ebselen oxide to block alginate secretion was also observed for strains containing Alg44-C98A or C98S mutation (Kim et al., 2021). This suggests that Ebselen oxide may exhibit its anti-biofilm effects by targeting additional proteins involved in alginate biogenesis. This is not surprising given that Ebselen oxide has previously been shown to also inhibit diguanylate cyclase activity (Lieberman et al., 2014). Given the importance of alginate production in *P. aeruginosa* infections of cystic fibrosis patients and the central role of c-di-GMP binding to Alg44 for alginate biosynthesis, inhibitors that are selectively able to block this binding interaction could prove useful as therapeutics to treat mucoid *P. aeruginosa* infections.

### Inhibitors That Bind and Sequester c-di-GMP

An alternative approach to blocking c-di-GMP signaling would be to use molecules that specifically bind and sequester c-di-GMP inside the cell. This approach avoids issues involving the redundancy of targeting the various DGCs and PDEs, which vary between bacterial species, by directly focusing on binding to c-di-GMP. In 2016, Herman Sintim’s lab reported the first example of inhibiting c-di-GMP processing using a small molecule intercalator, proflavine, to induce the supramolecular polymerization of c-di-GMP (Nakayama et al., 2016). This sequestration of c-di-GMP into supramolecular polymers inhibited its degradation by PDE enzymes.

In 2020, Stephan Grzesiek and Urs Jenal proposed an alternative approach to block biofilm formation also through the sequestration of c-di-GMP (Hee et al., 2020). They previously showed that a short arginine rich peptide present in a novel family of chemotaxis protein Y (CheY)-like proteins binds to c-di-GMP with nanomolar affinity (Nesper et al., 2017). From the NMR structure of this protein domain, they were able to design a minimal 36-aa peptide sequence with low nanomolar binding affinity for c-di-GMP and specificity for c-di-GMP over other related cyclic di-nucleotides (Hee et al., 2020). Recombinant expression of this peptide as a maltose binding protein fusion in *P. aeruginosa* resulted in significant inhibition of biofilm formation and even showed the ability to disrupt pre-formed biofilms. This work provides a proof of concept that peptide-based c-di-GMP binders could prove useful as anti-biofilm agents; however, there is still significant work required to translate this into a therapeutic. It must be shown that these peptides can be delivered to bacterial cells in sufficient quantities to effectively sequester c-di-GMP, and that the peptides are metabolically stable enough to function *in vivo*.

## Discussion and Outlook

In this review, we have discussed the importance of the second messenger, c-di-GMP, in bacterial biofilm formation, and its role in activating the biogenesis of biofilm exopolysaccharides. A tremendous amount of structural and mechanist work over the past decade has begun to shed light into the biochemical mechanisms by which c-di-GMP binding is able to post-translationally activate the synthase enzymes involved in the biosynthesis of cellulose, alginate, Pel, and PNAG polysaccharides that are common components of bacterial biofilm EPS. Although the biosynthesis of all four of these polysaccharides is directly regulated by c-di-GMP, they each use different c-di-GMP receptors and mechanism of activation to accomplish this regulation.

The cellulose synthase complex responsible for the biosynthesis of bacterial cellulose is by far the most well studied of these pathways. In this system, c-di-GMP binds directly to the PilZ domain of the BcsA protein (Morgan et al., 2013, 2014) inducing a conformational change in the “gating loop” to facilitate binding of UDP-Glc to the active site of the glycosyltransferase domain. The recent discovery that a second protein present in the *E. coli* cellulose synthase complex, BcsE, also binds to c-di-GMP (Fang et al., 2014) and significantly increases the *in vivo* production of pEtN cellulose secreted by this organism (Thongsomboon et al., 2018), suggests that the mechanism of c-di-GMP activation of cellulose biogenesis may be more complex than initially thought. One hypothesis is that c-di-GMP binding to BcsE may function to shuttle c-di-GMP from the cytosol to the PilZ domain of BcsA for efficient cellulose polymerization (Zouhir et al., 2020). An alternative hypothesis is that c-di-GMP binding to BcsE may serve to activate a BcsEFG complex to catalyze the transfer of pEtN groups onto the growing cellulose polysaccharide (Sun et al., 2018; Thongsomboon et al., 2018; Anderson et al., 2020). This second hypothesis is intriguing as c-di-GMP would have a dual function in both activating cellulose biogenesis and its modification with pEtN. Recent cryo-EM structures of the entire intact *E. coli* inner membrane cellulose synthase complex are beginning to shed more light into this mechanism (Abidi et al., 2021; Acheson et al., 2021), but clearly more mechanistic studies are required to work the exact role of c-di-GMP in cellulose biogenesis.

The biosynthesis of alginate in *P. aeruginosa* is also post-translationally regulated by binding of c-di-GMP to the PilZ domain of the alginate co-polymerase protein Alg44 (Merighi et al., 2007). Together Alg44 and Alg8 form an inner membrane glycosyltransferase complex that polymerizes GDP-ManA to form alginate. Pel polysaccharide biogenesis is activated by c-di-GMP in a similar way through binding to PelD (Lee et al., 2007). PelD then interacts directly with PelE, PelF, and PelG to form an inner membrane glycosyltransferase complex responsible for both the polymerization and secretion of Pel (Whitfield et al., 2020b). The exact mechanism by which c-di-GMP binding activates the Alg44/Alg8 and PelDEFG complexes is still not known, but crystal structures of Alg44 and PelD suggest that c-di-GMP binding may induce a conformational change in these proteins that could be responsible for activating their respective complexes (Whitney et al., 2012, 2015). The biogenesis of PNAG uses a different mechanism of c-di-GMP activation altogether. In that system, c-di-GMP binds to both PgaC and PgaD to stabilize an inner membrane complex between the two proteins, allowing for PNAG biosynthesis to occur (Steiner et al., 2013). The exact mechanism by which c-di-GMP binds and activates the PgaCD complex still needs to be worked out.

It is clear that c-di-GMP binding is critical for the biogenesis of cellulose, alginate, Pel and PNAG polysaccharides, and is required for biofilm formation numerous human pathogens. More detailed structural information on exactly how c-di-GMP binding activates the protein complexes responsible for biogenesis of these polysaccharides will be critical for efforts to develop new anti-biofilm agents to block the biosynthesis of these polysaccharides. Recent work by Vincent Lee, Stephan Grzesiek, and Urs Jenal has shown that it is possible to use small molecules that inhibit the binding of c-di-GMP to Alg44 (Zhou et al., 2017; Kim et al., 2021), or to sequester intracellular c-di-GMP using c-di-GMP binding peptides (Hee et al., 2020) to block alginate secretion and biofilm formation in *P. aeruginosa*. These are still early proof of concept studies and more work is needed to evaluate the potential cytotoxicity of these compounds and their ability to block biofilm formation in animal models of *P. aeruginosa* infection. Thus, unraveling the mechanistic details of c-di-GMP activation of biofilm exopolysaccharide biosynthesis may well lead to the development of new therapeutic approaches to directly target biofilm formation.

## Author Contributions

LK drafted the manuscript. MP and LK were responsible for preparing the figures in the manuscript. Both authors contributed to the final writing of the manuscript and approved the submitted version.

## Conflict of Interest

The authors declare that the research was conducted in the absence of any commercial or financial relationships that could be construed as a potential conflict of interest.

## Publisher’s Note

All claims expressed in this article are solely those of the authors and do not necessarily represent those of their affiliated organizations, or those of the publisher, the editors and the reviewers. Any product that may be evaluated in this article, or claim that may be made by its manufacturer, is not guaranteed or endorsed by the publisher.
